# A Meta-analysis of Attachment to Parents and Delinquency

**DOI:** 10.1007/s10802-011-9608-1

**Published:** 2012-01-27

**Authors:** Machteld Hoeve, Geert Jan J. M. Stams, Claudia E. van der Put, Judith Semon Dubas, Peter H. van der Laan, Jan R. M. Gerris

**Affiliations:** 1 Research Institute Child Development and Education, University of Amsterdam, P.O. Box 94208, 1090 GE Amsterdam, The Netherlands; 2 Department of Developmental Psychology, Utrecht University, Utrecht, The Netherlands; 3 Netherlands Institute for the Study of Crime and Law Enforcement (NWO-NSCR), Amsterdam, The Netherlands; 4 Department of Criminology, VU University Amsterdam, Amsterdam, The Netherlands; 5 Institue of Family and Child Care Studies, Radboud University Nijmegen, Nijmegen, The Netherlands

**Keywords:** Attachment, Delinquency, Sex-differences, Age effects, Meta-analysis

## Abstract

To investigate the link between attachment to parents and delinquency, and the potential moderating effects of age and sex, 74 published and unpublished manuscripts (*N* = 55,537 participants) were subjected to a multilevel meta-analysis. A mean small to moderate effect size was found (*r* = 0.18). Poor attachment to parents was significantly linked to delinquency in boys and girls. Stronger effect sizes were found for attachment to mothers than attachment to fathers. In addition, stronger effect sizes were found if the child and the parent had the same sex compared to cross-sex pairs of children and parents. Age of the participants moderated the link between attachment and delinquency: larger effect sizes were found in younger than in older participants. It can be concluded that attachment is associated with juvenile delinquency. Attachment could therefore be a target for intervention to reduce or prevent future delinquent behavior in juveniles.

Poor attachment to parents is considered to be one of the causes of delinquency (Bowlby 1944; Hirschi 1969). Although many empirical studies found evidence suggesting that poor attachment to parents increases the risk of delinquent behavior, a systematic review of the link between attachment and delinquency has not been conducted yet. It seems important to know how strong attachment is associated with delinquency in males, females and various age groups, as this knowledge can be used to develop and improve intervention programs that target delinquency in youth. We therefore seek to integrate results from empirical studies examining the association between attachment and delinquency by means of a meta-analysis.

## Attachment and Delinquency

Two main theories addressing the relation between attachment and delinquency are the social control theory (Hirschi 1969; Sampson and Laub 1993) and attachment theory (Ainsworth 1979; Bowlby 1973). The first theory is a criminological theory that was developed by Hirschi (1969), who conceptualized attachment as an affective bond through which children internalize conventional norms of society. According to Hirschi, delinquency will be low in families with strong affective ties, because juveniles who are strongly attached to their parents are more likely to care about the normative expectations of their parents, which protects against delinquent impulses. The quality of attachment functions as an *indirect parental control*: conventional behavior of the child is achieved as a by-product of strong child-parent attachments (Hirschi 1969). Delinquent behavior, however, will increase if the bond to the parent is weak.

The second main theory that addresses the link between attachment and delinquency is attachment theory. Observing mothers and their infants, Bowlby (1973) identified an intense distress experienced by infants who had been separated from their parents. These children were crying and clinging to their mother when they were to be separated. Bowlby called these behaviors attachment behaviors, which are natural adaptive responses to separation that serve an evolutionary function. If the parent-child attachment relationship is disrupted during infancy, long-term negative consequences are the inability to show affection or concern for others and aggressive and delinquent behavior (Bowlby 1944, 1973).

Over the years, scholars began to investigate attachment relationships between adolescents and their parents and attachment relationships in young adulthood (e.g., Ainsworth 1989), focusing on *attachment representations* rather than observing attachment behaviors in infants. Bowlby (1988) believed that from infancy, children internalize and organize patterns of relating to people. They can mentally represent their experiences with attachment figures and construct ideas and expectations about relationships with primary as well as secondary attachment figures. As such, an internal working model of attachment is gradually shaped during development (Bretherton 1990). Next to observational methods that measure attachment in early childhood in terms of behavioral responses to caregivers, questionnaires and (in-depth) interview methods were developed to measure attachment representations in middle childhood (e.g., Dwyer 2005; Kerns et al. 2000), adolescence (Allen and Land 1999) and (young) adulthood (Bretherton 1990).

In sum, attachment theory is a theory of both normal and abnormal development that focuses on the impact of parent-child attachment relationships on healthy development and psychopathology, including juvenile delinquency (Sroufe et al. 1999). Control theory is a theory of abnormal development that mainly focuses on explaining criminal behavior on the basis of social control. Essential for control theory is the affectional bond that the child forms to his or her parents, which is thought to influence child behavior through the psychological presence of the parent. Whereas an affectional bond is a necessary but not a sufficient condition for attachment in attachment theory (see Schuengel and Van IJzendoorn 2001), it is both a necessary and sufficient condition for attachment in control theory. Despite these differences between attachment theory and social control theory, it is safe to hypothesize that poor or disturbed attachment is associated with risk for delinquency.

Several previous meta-analyses have been conducted that focused on the link between insecure attachment relationships and problem behavior in children. For example, Van IJzendoorn et al. (1999), analyzing 12 studies, found a significant link between disorganized attachment and externalizing problem behavior. Also, in a more recent meta-analysis it was found that attachment insecurity was significantly linked with externalizing behaviors in 69 studies (Fearon et al. 2010). Unfortunately, these meta-analyses did not differentiate between the two main types of externalizing problems, that is, aggression and delinquency (e.g., Achenbach 1991). Given the significance of delinquency among adolescents and young adults as a problem in society, we exclusively focus on delinquency in this study. Previous research has shown that delinquency and aggression are conceptually distinct types of behaviors. For example, the trajectories of aggression and delinquency have been found to show a different developmental pattern (Bongers et al. 2004). Furthermore, different risk factors have been found for aggressive and delinquent behavior (Dishion and Patterson 2006). Therefore, it is seems important to conduct a separate meta-analytic study of the association between attachment and delinquency, which enables studying a number of specific moderators of the association between attachment and delinquency, such as the source of information on delinquency, and perhaps most importantly, the concept of attachment.

## Age Effects

To what extent the link between attachment and delinquency changes over the life course is unclear. At least two rival hypotheses concerning the attachment-delinquency link over time can be drawn. Static theories postulate that the variation in criminal behavior is explained by individual differences in latent criminal propensity, and that these individual differences remain constant over time (Ezell and Cohen 2005). Hirschi and Gottfredson (2001, p. 229) even state that ‘identification of the causes of crime at one age may suffice to identify them at other ages as well – if so, cohort or longitudinal studies of crime are unnecessary’. Thus, according to this hypothesis the link between attachment to parents and delinquency should be similar across late childhood, adolescence and emerging adulthood.

Dynamic developmental theories assume that change is possible. For example, Sampson and Laub’s (2005) age-graded social control theory assumes that changes in life circumstances may generate turning points in an individual’s criminal career. Thus, in contrast to static theories, dynamic theories postulate that life circumstances are related to criminal behavior and that crime can be modified over the life course. According to Sampson and Laub (2005), delinquent behavior is inhibited during childhood and adolescence by bonds to the family and school. During (young) adulthood, social ties to labor or marriage and turning points in life can modify trajectories of criminal offending. Based on dynamic theories, such as that of Sampson and Laub, the influence of family ties should diminish in late adolescence and young adulthood as the individual makes other important social ties. Thus, while static theories, such as the theory of Hirschi, assume that the attachment-delinquency link is independent of age, dynamic models such as the age graded theory of Sampson and Laub, state that the attachment-delinquency link is strongest during childhood and gradually decreases during adolescence and early adulthood.

## Sex-differences

It was not until feminist criminologists criticized mainstream theories for exclusively focusing on criminal behavior of males (Daly and Chesney-Lind 1999) that scholars began to investigate potential explanations of sex differences in delinquency, referred to as the gender gap. Feminist scholars argued for gender sensitive theories of crime (Miller and Mullins 2009). According to control theorists, processes that enhance or prevent delinquency are gender neutral, and the gap in delinquency between males and females can be explained by differences in the quality of bonds to parents (Agnew 2009). Social ties to parents are stronger in females than in males, which explains why females’ levels of delinquency are lower than those of males.

Some scholars disagree on whether sex-differences exist in attachment relationships. According to Del Giudice (2009), sex-differences exist in the quality of the attachment relationship, yet secure attachments are similarly often found in males and females. In a nutshell Del Giudice states that females are more vulnerable to develop anxious-ambivalent patterns of attachment, while males are expected to adopt the avoidant attachment type. As a consequence, males are more likely to adopt competitive and aggressive traits and externalizing problems, such as delinquency, whereas females are more likely to develop internalized problems, such as anxiety and depression (Del Giudice 2009). However, Bakermans-Kranenburg et al. (2005) criticized Del Giudice’s model for lack of empirical evidence. Their meta-analyses did not support the hypothesis of sex-differences in attachment.

Studies of sex-differences in the link between attachment and delinquency are scarce and their findings are contradictory. Some studies reported stronger effects of attachment in girls (e.g. Nye 1958), whereas others concluded that the family bond is more important to boys. For example, it was found that family strain (Hay 2003) and the quality of parental caregiving (Rothbaum and Weisz 1994) had stronger effects on males than on females. Insecure attachment was more strongly linked to externalizing behavior in samples with boys than in samples with girls (Fearon et al. 2010). A third group of researchers found very few sex-differences in family related factors of delinquency (Hubbard and Pratt 2002; Loeber and Stouthamer-Loeber 1986). Moffitt et al. (2001) extensively investigated potential sex-differences in the prevalence of risk factors and the impact of family risk factors on delinquency and concluded that, in general, boys seem to be more exposed to risk factors of delinquency, rather than that they are more vulnerable for risk factors of delinquency compared to girls. Thus, findings as to whether there are sex differences with regard to the link between attachment and delinquency have been inconsistent and a meta-analysis could shed light on this issue.

Apart from potential differences between males and females in the link between attachment and delinquency, differences may appear depending on the sex of the parent for several reasons. First, the *quantity* of the time fathers and mothers spend with their children is different. In general mothers spend more time in taking care of their children (Dubas and Gerris 2002), which could result in stronger ties to mothers and a stronger effect of the quality of the mother-child bond on the child’s behavior. Second, there are indications that parental involvement is also *qualitatively* different (Videon 2005). Offending behavior by the father predicts delinquent behavior of their sons (Farrington et al. 2001). Moreover, the longer antisocial fathers live with their families the higher the risk for their children’s antisocial behavior (Jaffee et al. 2003). A possible explanation for this finding is that attachment relationships to these fathers are more problematic. Attachment relationships are influenced by the extent to which the parent is sensitive to the needs of the child (De Wolff and van IJzendoorn 1997). Although fathers and mothers have been found to be equally capable of sensitive responding, fathers tend to exercise their sensitivity less often (Lamb 1982). This is essential since delinquency is more common among boys than among girls. Despite this possibility, both control theory and attachment theory are not very clear about the effects of attachments to fathers. Moreover, relatively little research has examined the quality of attachment relationships to fathers compared to attachment to mothers in relation to the child’s delinquent behavior (Williams and Kelly 2005). Finally, it is unknown whether the consequences of insecure attachment relationships or poor bonds to parents with regard to delinquency gradually change over time and whether this potential change is different for boys and girls.

## The Present Study

Many studies found evidence to suggest that poor attachment relationships to parents increase the risk of delinquent behavior. The inconsistencies in the literature, however, make it difficult to summarize the results in a narrative review. Previous studies did not summarize potential sex-differences in the link between attachment and delinquency, and it is therefore unknown whether the attachment-delinquency link is gender-specific or neutral. Given that studies examining attachment to mothers and fathers separately are scarce, it remains unclear whether attachment to father has a different effect on delinquency in sons and daughters than attachment to mother. Finally, previous studies have not systematically investigated whether the attachment-delinquency link varies by age, that is, whether this link is stronger in younger children than in adolescents and early adults.

The aim of the present meta-analytic study is therefore to examine the extent to which attachment is related to delinquency and to gain insight into sex and age differences. This study could advance theories on attachment and delinquency, particularly with regard to potential conceptual differences of attachment constructs in social control and attachment theories, potential gender differences (debate on whether gender specific theories are necessary) and age differences (static versus dynamic theories). It may also provide starting points for developing or improving interventions for delinquent youth or children at risk for delinquency.

The present meta-analytic study addresses the following research questions: Is poor attachment significantly associated with high levels of delinquency? How strong is the association between attachment and delinquency? Is age a moderator of the attachment-delinquency link? Is the association between attachment and delinquency moderated by sex of the child and the parent? We also test whether the attachment-delinquency link depends on the theoretical background of the study (control theory or attachment theory) and measure of attachment (observation, self-report, parent report or multi-method), and whether conceptual differences in attachment constructs (presence of parental control items) influence the relation between attachment and delinquency. Further, we examine moderator effects of methodological characteristics (sample size, publication status, study design and delinquency source) in order to investigate potential influences of study quality on effect size. We also address the relative importance of the moderators and interaction effects of sex and age in a multivariate model.

## Method

### Sample of Studies

For the selection of studies four criteria were formulated. First, studies in which delinquency was defined as behavior prohibited by the law were selected. Studies that focused exclusively on problem behavior, which we consider as behavior that is not prohibited by the law, were not included. Second, studies had to focus on the quality of the attachment relationship, the bond to parents, or affectional identification, which considers both the child and the parent but not specific behavior of the child towards the parent or vice versa (e.g., parenting). If attachment was operationalized exclusively as behavior of the parent towards the child (e.g., parental supervision) the study was not selected. Also, studies on non-parent attachment relations such as attachments to peers were excluded. We collected empirical studies that tested assumptions from social control theory as well as from attachment theory. Third, samples would have to include participants from Western countries, because it is known that cultural differences exist with regard to child-rearing and attachment (e.g., Van IJzendoorn and Kroonenberg 1988). Finally, manuscripts had to present the results of a bivariate analysis of the association between attachment and delinquency or provide enough details to calculate a bivariate test statistic.

In spring 2010, studies were collected according to the following procedure. First, electronic databases such as ERIC, PsycINFO, Sociological Abstracts, and Criminal Justice Abstracts, were searched through for articles, books, chapters, paper presentations, dissertations and reviews. Our purpose was to find as many studies as possible, and therefore we used a variety of terms related to attachment and delinquency. Given that social bonds are not limited to bonds to parents in control theory, we used terms related to parent. Search terms such as delinq*, crim*, offend*, anti-social were cross-referenced with attachment, bond*, parent-child relation*, and social control: (“social control” or attachment or bond*) and (delinq* or crim* or antisocial) and (parent*). After that, manual searches were applied, which means that reference lists of reviews and other articles were checked in order to find relevant studies not found in the electronic databases. We found 346 records in the databases of which we selected 137 on the basis of the information in the abstract. We finally found 74 manuscripts that met our criteria, which reported 151 analyses on the attachment-delinquency link. Findings of 63 independent studies that focused on a total of 55,537 subjects were reported.

### File Drawer Problem

The tendency of journals to accept papers that report strong significant associations, referred to as publication bias, may have implications for the final conclusions of the meta-analysis (Rosenthal 1991; Van IJzendoorn 1998). Rosenthal (1979) identified this problem as the file drawer problem. Several methods exist to address potential effects of publication bias, but each has its own shortcoming (Rothstein 2008). The best solution to this difficulty is to try to prevent effects of publication bias and obtain all unpublished material as best as possible (e.g., Mullen 1989; Rosenthal 1991). Therefore, the present meta-analysis includes unpublished studies. We considered journal articles, books and book sections as published and dissertations as unpublished. We found 14 unpublished manuscripts: 13 dissertations and 1 paper presentation.

Following the advice of Rothstein (2008), we apply two of the conventional methods that address publication bias. First, we provide a fail-safe number, which estimates the number of unretrieved studies averaging null results needed to bring the overall combined effect size at a non-significant level (Rosenthal 1991). A second method of examining what effect publication bias could have on the meta-analytic results is inspecting the distribution of each individual study’s effect size on the horizontal axis against its sample size, standard error or precision (the reciprocal of the standard error) on the vertical axis. The distribution of effect sizes should be shaped as a funnel if no publication bias is present, since the more numerous studies with small sample sizes are expected to show a larger variation in the magnitude of effect sizes than the less numerous studies with large effect sizes. A violation of funnel plot symmetry reflects publication bias, that is, a selective inclusion of studies showing positive or negative outcomes (Sutton et al. 2000). In the present study, funnel plot asymmetry was tested by regressing the standard normal deviate, defined as the effect size divided by its standard error, against the estimate’s precision (the inverse of the standard error), which largely depends on sample size (Egger et al. 1997). If there is asymmetry, the regression line does not run through the origin and the intercept significantly deviates from zero.

### Coding of the Study Outcomes and Characteristics

We focused on those study characteristics that were required to test our hypotheses. For the coding of the study characteristics we applied the system that we developed for a previous meta-analysis on parenting and delinquency (Hoeve et al. 2009). First, we retrieved the study results (test statistic and value) and sample size. With regard to age of the participants, we coded age of the subjects at the time of the attachment measurement (ages 6.4–38.3) and age of the subjects at the time of the delinquency measurement (ages 7.4–38.3), as these ages differed in longitudinal studies. Sex was coded as follows: percentage of females in the sample (0–100%, *M* = 43.1) and sex of the parent (father, mother, both parents or not specified). Given that unpublished studies are not verified by an outside public and are generally of poorer quality than published studies, we analyzed whether the quality influenced the combined effect size. We therefore gathered data on publication status (i.e., published or not) and some quality indicators from the empirical studies: sample size (63 to 12,604), design (cross-sectional, longitudinal or retrospective), source of delinquency (self-reported, official, or more than one source). Finally, we collected data on ethnicity (the percentage of non-Caucasian or nonindigenous subjects in the sample; 0–100%, *M* = 37.6) because research has found this background variable to be linked to delinquency (e.g., Farrington et al. 2003; Junger-Tas 1997).

In order to detect potential effects of conceptual differences between the studies concerning the concept of attachment, we first retrieved information on the theoretical background of the study: theoretical model (control theory, attachment theory, or other). Second, we collected data on the measure of attachment (observation, AAI, self-report, parent report, or multi-method), because at least five very different methods of measuring attachment are used, including 1) observation, which is typically applied to collect information on attachment relations of infants (e.g., the Strange Situation procedure of Ainsworth, 1979), 2) interview methods that measure attachment representations, such as the Adult Attachment Interview (AAI; George et al. 1996), 3) self-report measures, such as the Inventory of Parent and Peer Attachment (IPPA; Armsden and Greenberg 1987), 4) parent report, such as a scale derived from the Hudson’s Index of Parental Attitudes and the Child’s Attitude Toward Mother (Father) Scale (Hudson 1982), and 5) multi-method (e.g., parent and self-report). The studies that met our inclusion criteria concentrated on current attachment relations (observation and self-report, parent report and multi-method studies) and attachment representations (AAI studies). Finally, given that studies that tested Hirschi’s theory of social control sometimes included parental control items, we coded whether or not the measure of attachment in each study included parental control items, such as parental supervision, rules setting, discipline techniques (parental control versus no parental control items).

The first author coded the effect sizes and study characteristics. Reliability of the coding scheme was checked by having a subset of the study characteristics coded by a master student. Forty five analyses were randomly selected of which 11 analyses were used for training purposes. Inter-rater agreement was analyzed for each of the study outcomes and characteristics of 34 analyses by calculating the percentage of agreement for all study characteristics, Kappa for categorical variables and intraclass correlation for interval and ratio variables. The inter-rater reliability appeared to be substantial to perfect with Kappas ranging from 0.78 (94% agreement) for parental control to 1.00 for publication status and gender of the child (both 100% agreement) and intraclass correlations ranging from 0.95 (70% agreement) for the effect size (value) to 1.00 for age at delinquency measurement (79% agreement), age at attachment measurement (85% agreement), ethnicity (91% agreement), and percentage of females (97% agreement).

### Analysis

For each study an effect size was calculated. We used Pearson’s *r* to express the link between attachment and delinquency. We used the formulas of Mullen (1989) and Lipsey and Wilson (2001) to transform test statistics into correlation *r*. Each correlation was transformed to a Fisher’s *Z* before combined effect sizes were calculated and moderator analyses were conducted (Mullen 1989). We checked for outliers on the basis of standardized *z*-values larger than 3.29 or smaller than -3.29 (Tabachnick and Fidell 1989). No outliers were identified. We centered each continuous moderator variable around its mean and for the categorical variables we made dichotomous dummy codes.

After examining the extent of the variation in effect sizes, we conducted a test for homogeneity of effect sizes (Rosenthal 1991). Independence of study results is desirable when conducting a meta-analysis in order to preclude that a particular study is weighted more strongly than the others (Lipsey and Wilson 2001; Mullen 1989; Rosenthal 1991).

To deal with dependency of study results, we used a multilevel random effects model for the calculation of combined effect sizes and moderator-analyses (Hox 2002; Van den Noortgate and Onghena 2003). We used the program MLwiN for conducting multilevel analysis and used an adapted set up, described by Hox (2002) to make our models suitable for meta-analysis. A multilevel random effects model accounts for the hierarchical structure of the data, in which the effect sizes or study results (the lowest level) are nested within studies (the highest level). In multilevel research, a random-effects model is often used, which can be extended by including moderators. Iterative maximum likelihood procedures are applied to estimate unknown parameters. The intercept only model (without moderators) is equivalent to the random-effects model of Hedges and Olkin (1985). In the complete model covariates can be added to test for potential moderators. Van den Noortgate and Onghena (2003) compared multilevel meta-analysis with traditional meta-analytic methods and concluded that maximum likelihood multilevel approach is in general superior to the fixed-effects approaches and that the results of the multilevel approach are not substantially different from the results of the traditional random-effects approaches for intercept only models.

## Results

### Central Tendency and Variability

The overall mean effect size for the association between attachment and delinquency was significant (*r* = 0.18, *p* < 0.001, *k* = 151), indicating that youth with poor attachment relationships have higher levels of delinquency (see Table 1). A homogeneity test revealed that effect sizes were heterogeneous (*Z* = 6.0, *p* < 0.001). Thus, although there was a significant association between most of the parenting variables and delinquency, this was not a consistent phenomenon. This suggests that study outcomes are likely influenced by study characteristics and justifies the investigation of potential moderators, which we report in the next subsections. The fail-safe number based on aggregated independent effect sizes (*p* = 0.05, *k* = 63) was 28,381. Given that the fail-safe number exceeded the critical value 5 *k* + 10, as suggested by Rosenthal (1991), no possible file drawer problems were indicated (28,381 > 765). We also examined possible publication bias by testing funnel plot asymmetry. The standard normal deviate was regressed against the estimate’s precision. As the intercept did not significantly deviate from zero (*t* = 0.666, *p* = 0.508), there was no indication of funnel plot asymmetry and therefore no indication of publication bias. These findings suggest that the mean effect size can be considered robust.

**Table 1:** Results for the overall mean effect size and discrete moderators (bivariate models)

Moderator variables	# Studies	# ES	Mean r	Z	Heterogeneity	Δfit
Overall	61	149	0.18	12.3***	6.0***	-
*Concept of Attachment*						
Theoretical background					4.0***	0.04
Control Theory (RC)	45	107	0.18	-		
Attachment Theory	11	18	0.20	0.4		
Other/ unknown	14	26	0.17	-0.5		
Measure of attachment					4.3***	83.3***
Self-report (RC)	51	126	0.16	-		
AAI	5	6	0.12	-0.7		
Parent-report	6	10	0.25	3.6***		
Multi-method	5	8	0.39	8.7***		
Parental control items					4.7***	4.7*
No	55	131	0.17	-		
Yes	7	17	0.29	3.2***		
*Sex*						
Sex of parent					4.0***	8.5*
Mother (RC)	27	44	0.21	-		
Father	22	35	0.19	-2.6**		
Both or not specified	38	72	0.17	-2.0*		
Sex of child and parent					2.1*	30.9***
Same sex (RC)	10	18	0.22	-		
Cross-sex	13	24	0.18	-2.1*		
*Methodological Moderators*						
Publication status					6.0***	20.3***
Published (RC)	52	129	0.19	-		
Unpublished	13	22	0.13	-4.5***		
Design					5.5***	5.9*
Cross-sectional (RC)	50	119	0.18	-		
Longitudinal	17	30	0.17	-0.9		
Retrospective	1	2	0.46	2.2*		
Delinquency Source						
Self-reported (RC)	53	128	0.19	-	4.3***	26.7***
Official record	11	16	0.10	-4.9***		
More than one source	6	7	0.21	0.6		

RC = reference category, # studies = number of independent studies; # ES = number of effect sizes, *Z* = difference in mean *r* with reference category, mean *r* = mean effect size (*r*), heterogeneity = within class heterogeneity (*Z*), Δfit = difference with model without moderators (χ^2^)

*p < 0.05; **p < 0.01; ***p < 0.001

### Concept of Attachment

Theoretical background of studies proved to be nonsignificant, indicating that the study results with regard to the link between attachment and delinquency were independent from disciplinary background (Table 1). However, studies resulted in different effect sizes depending on which method was used to measure attachment. Effect sizes were significantly larger for studies using parent reports (*r* = 0.25 for parent report versus *r* = 0.16 for self-report; *Z* = 3.6, *p* < 0.001; Table 1) and various methods to measure attachment (*r* = 0.39 for parent report versus *r* = 0.16 for self-report; *Z* = 8.7, *p* < 0.001; Table 1) than studies using self-report methods. In addition, if studies used a measure that included parental control items next to attachment relationship items, the attachment-delinquency link was significantly stronger compared to studies that focused exclusively on the attachment relationship (*r* = 0.17 for measures without parental control items versus *r* = 0.28 with parental control items; *Z* = 3.2, *p* < 0.001; Table 1).

### Age

We found a trend for the moderating effect of average *age of the child* at which *attachment data* were collected (see Table 2 for continuous moderators). A negative association between average age and effect size was found, indicating that studies with younger participants showed a stronger link between attachment and delinquency (*Z* = -2.0, *p* < 0.05). With regard to the age of the subject at which *delinquency data* were collected we found a significant moderating effect. Studies that measured delinquency in younger participants resulted in larger effect sizes than studies in older participants (*Z* = -9.5, *p* < 0.001). Thus, in general the link between attachment and delinquency becomes weaker as children age.

**Table 2:** Results for the continuous moderators (bivariate models)

Moderator variables	# ES	β_0_ (SD)	β_1_ (SD)	Z	etero-geneity	Δfit
Sample characteristics						
Percentage females	148	0.182 (0.015)	-0.00005 (0.00015)	-0.4	4.0***	1.3
Ethnicity	120	0.184 (0.018)	-0.00075 (0.00035)	2.1*	5.0***	15.6***
Age Attachment	146	0.180 (0.015)	-0.00700 (0.00300)	-2.0*	5.5***	1.2
Age Delinquency	148	0.179 (0.018)	-0.01900 (0.00200)	-9.5***	4.8***	88.8***
Methodological moderator						
Sample size	151	0.189 (.015)	-0.00000 (0.00000)^a^	-3.3***	6.0***	11.0***

Ethnicity = Percentage of non-Caucasian or nonindigenous participants. Age Attachment = Age at attachment measurement, Age Delinquency = Age at delinquency measurement, # ES = number of effect sizes, *Z* = significance of moderator, heterogeneity = within class heterogeneity (*Z*), Δfit = difference with model without moderators (χ^2^)

^a^-0.0000064 (0.0000019)

*p < 0.05; ***p < 0.001

### Sex of the Child and the Parent

Sex of the child did not moderate the attachment-delinquency association. (see Table 2). However, attachment to mothers was more strongly related to delinquency than attachment to fathers (*r* = 0.21 for mothers versus *r* = 0.19 for fathers; *Z* = -2.6, *p* < 0.01). Also, studies that focused on mothers alone yielded larger effect sizes than studies that focused on both parents or on ‘a parent’ (*r* = 0.19 for mothers versus *r* = 0.17 for both parents or parent not specified; *Z* = -2.0, *p* < 0.05). In order to analyze whether parents had more influence on a child with the same sex, we created a variable with the categories: (1) fathers - boys and mothers - girls, (2) fathers - girls and mothers -boys. The attachment-delinquency link was significantly stronger if parent and child had the same sex (*r* = 0.22 for same sex versus *r* = 0.18 for different sex; *Z* = -2.1, *p* < 0.05).

### Methodological Moderators

We found that sample size was a significant moderator (*Z* = -3.3, *p* < 0.001; Table 2). Studies with smaller sample sizes showed a larger effect size than studies with larger numbers of subjects. We found significant differences between published and unpublished studies (*r* = 0.19 for published studies versus *r* = 0.13 for unpublished studies; *Z* = -4.5, *p* < 0.001; Table 1), which is in accordance with previous findings with regard to publication bias (Van IJzendoorn 1998). Cross-sectional and longitudinal studies resulted in relatively similar links between attachment and delinquency. We analyzed whether delinquency source was associated with effect size. Studies that relied on self-report measures of delinquency yielded larger effect sizes than studies in which delinquency data was collected via official records, such as police or court records (i.e., arrests or convictions; *r* = -0.19 for self-reported delinquency versus *r* = -0.10 for official delinquency; *Z* = -4.9, *p* < 0.001; Table 1). Ethnicity was significantly linked to effect size (*Z* = 2.1, *p* < 0.05; Table 2), indicating that studies with more nonindigenous or non-Caucasians in their sample resulted in larger effect sizes. Almost all bivariate models, in which the association between one study characteristic and study outcome is analyzed, have a significantly better fit than the intercept only model without explanatory variables (see Tables 1 and 2).

### Multivariate Analyses

Finally, two multivariate analyses were conducted in order to analyze the unique contribution of sex of the child and age on the attachment-delinquency link, over and above methodological and background moderators. We analyzed separate models for age of participants at the time of the measurement of attachment and at the time of the measurement of delinquency, as in longitudinal studies these ages were different. The models included percentage of females, age of the child, and the interaction of sex and age and the remaining moderators that were significantly linked to effect size in the bivariate analyses. No effects of sex (percentage of females) were found (Table 3). In the models we found a significant effect and a trend toward the effect of age (*Z* = -1.3, *p* < 0.10, for age at attachment measurement, and *Z* = -2.4, *p* < 0.01, for age at delinquency measurement). No significant interaction effects were found for age and sex of the child. With regard to the remaining moderators we found that sample size and study design was now nonsignificant in Model 2 and a trend was found toward an effect of ethnicity (Table 3). The multivariate models have a significantly better fit than the intercept only model without explanatory variables (Table 3).

**Table 3:** Results for the multivariate models

Moderator variables	Model 1		Model 2	
	β (SD)	Z	β (SD)	Z
Intercept	0.218 (0.027)	8.1***	0.186 (0.026)	7.2***
Control variables				
AAI (vs. self-report)	-0.054 (0.098)	-0.6	-0.115 (0.094)	-1.2
Parent report (vs. self-report)	0.129 (0.029)	4.4***	0.127 (0.029)	4.4***
Multi-method (vs. self-report)	0.224 (0.042)	5.3***	0.109 (0.048)	2.3*
Parental control (vs. no control items)	0.203 (0.047)	4.3***	0.191 (0.046)	4.2***
Sample size	- 018 (007)^a^	-2.6**	- 004 (007)^a^	-0.6
Published (vs. unpublished)	-0.550 (0.049)	-1.1***	-0.077 (0.046)	-1.7*
Longitudinal (vs. cross-sec)	-0.153 (0.036)	-4.3***	-0.031 (0.037)	-0.8
Retrospective (vs. cross-sectional)	000 (000)^a^	0.0	000 (000)^a^	0.0
Official (vs. self-reported)	-0.115 (0.026)	-4.4***	-0.118 (0.025)	-4.7***
More sources (vs. self-reported)	-0.144 (0.030)	-4.8***	-0.140 (0.030)	-4.7***
Ethnicity	- 772 (476)^a^	-1.6+	- 637 (464)^a^	-1.4+
Main effects				
Age	-0.008 (0.006)	-1.3+	-0.012 (0.005)	-2.4**
Percentage females	- 089 (231)^a^	-0.4	- 046 (231)^a^	-0.2
Interaction				
Age * Females	- 019 (107)^a^	-0.2	- 114 (103) ^a^	1.1
Heterogeneity – *Z*	4.8***		4.3***	
Δ fit - χ^2^ (10)	114.3***		135.4***	
*#* ES	116		116	

Model 1: Age = child’s age attachment measurement; Model 2: Age = child’s age at delinquency, *Z* = significance of moderator. Δfit = difference with model without moderators. *k* = number of analyses, # ES = number of effect sizes. ^a^0.000 removed in table

+ *p* < 0.10; *p < 0.05; **p < 0.01; ***p < 0.001

## Discussion

The present meta-analytic study summarizes and integrates previous findings on the link between attachment and delinquency, and examines factors that could have a moderating effect on this association, including age and sex. Results of this meta-analysis confirm previous findings in that poor attachment to parents is associated with more delinquent behavior. According to the criteria of Cohen (1988) for small, moderate and large effect sizes, the strength of the association between attachment and delinquency should be considered small to moderate (*r* = 0.18). This finding is relatively similar for studies examining the association between attachment and delinquency from the perspective of control theory and attachment theory.

Testing the potential effects of conceptual differences in attachment among studies revealed that not the theoretical background but the type of attachment measure moderated study outcomes. Studies that used parent reports and combined methods to measure attachment resulted in a relatively strong link between attachment and delinquency. This is consistent with findings of the meta-analysis by Fearon et al. (2010) showing that the relation between attachment and externalizing behavior problems was moderated by the type of attachment measure. A first explanation for this effect could be that type of attachment measurement is confounded with age. Particular attachment measures are typically used with subjects of specific ages, for example, behavioral observation for infants and attachment interviews (e.g., AAI) and self-report questionnaires for late adolescents and (young) adults. We found larger effect sizes with parent-report and mixed methods, which are commonly used with younger children, than with the AAI and self-report measures, which are used with late adolescents and (young) adults. However, given that both age and type of attachment measurement remained significant in the multivariate models, age cannot (entirely) explain the effect of measurement type.

Second, some scholars have argued that the type of attachment measure can have an effect on the study outcomes, because different attachment instrument may measure different attachment concepts (Van IJzendoorn and Bakermans-Kranenburg 2004). Parent reports of attachment and mixed methods had a stronger focus on the quality of the parent-child relationship and the bond between parent and child, but less on attachment security or internal working models of attachment. It should be noted that in particular security and internal working models of attachment are key concepts in the theory of attachment that was originally developed by John Bowlby. The most valid way to assess attachment in adolescents and (young) adults is considered to be the Adult Attachment Interview (AAI; Schuengel et al. 2011). It is in particular interesting to observe that (juvenile) delinquents in (forensic) institutional facilities show high rates of insecure and disorganized attachment (Zegers et al. 2008; Van IJzendoorn et al. 1997), which may have important consequences for treatment. Smith et al. (2010), for instance, showed that therapeutic alliance is affected by attachment (in) security. Schuengel and Van IJzendoorn (2001) considered attachment to be important in institutionalized treatment because the internal working model of attachment may affect quality of therapist-client relationships, treatment outcomes, and because institutionalization may have an effect in itself on attachment representations and future attachment behavior of the client.

Further, the combination of attachment and parental control, including supervision, rules setting and strictness had more impact on the child’s behavior than child-parent attachment relationships alone. Studies that used instruments to measure attachment, including parental control items, revealed larger effect sizes. This is not in line with the assumption of Hirschi (1969) and Nye (1958), who state that supervision and parental control is probably limited for adolescent children, because youths in this age period spend less time with their parents and are relatively autonomous. Given that most of the time parents are not able to supervise their children, direct parental control, such as supervision and discipline, are considered by control theorists as etiologically less relevant as a predictor of delinquency compared to indirect control, such as attachment (e.g., Hirschi 1969; Nye 1958). Our findings with regard to parental control do, however, not concur with our meta-analytic results on the link between parenting and delinquency, as we found moderate mean effect sizes for several types of parental control, including poor parental supervision (*r* = 0.23), and psychological control (*r* = 0.23; Hoeve et al. 2009). Possibly, attachment is more important at younger ages, while parental control remains to be an important risk factor of delinquency, given that the attachment-delinquency link was found to become weaker at older ages (current meta-analysis) while the parental control-delinquency link was not moderated by age (Hoeve et al. 2009). In conclusion, the supposition of control theory and attachment theory that disturbed attachment is linked to delinquency is supported by the current findings. However, in contrast to these theories, it seems that parental control and discipline are at least as or even more important for predicting delinquent behavior.

Effect sizes of more or less similar magnitude were found for males and females, indicating that poor bonds to parents similarly explain delinquency in both boys and girls. This finding may not refute the assertion of feminist criminologists that gender-sensitive theoretical models are needed to explain sex-differences in delinquency. Notably, the attachment-delinquency link may partly explain female delinquency, but does not provide an explanation as to why boys are more often involved in delinquent behavior than girls. Hagan (1999) explains this sex difference by pointing to the impact of gender-specific socialization processes within families that are related to differences in the distribution of power between the father and the mother. For example, in patriarchal families power relations between fathers and mothers are unequal. Such inequality may result in more parental control toward daughters than sons, which could offer an explanation for the gender gap in delinquency. It is also possible that different types of insecure attachment are related to different problem behaviors, as has been suggested by Del Giudice (2009). This is a promising future line of research.

In the present study, we found that attachment to mothers was more important for girls, while attachment to fathers was more important for boys with regard to the development of delinquency. Given that delinquency is more prevalent in boys, this finding has important implications for theory and practice. First, disturbed attachment to fathers should be recognized as a risk factor for delinquency in boys. Second, interventions that target delinquency in boys could profit from the involvement of fathers. This is in accordance with a recent study of Lundahl et al. (2008) showing that children benefited more if fathers attended a parent training compared to programs that focused on mothers only.

We found that the strength of the association between attachment and delinquency was negatively related to age, suggesting that the influence of attachment to parents on delinquency weakens as youngsters become older. These findings contradict the static perspective of Gottfredson and Hirschi (1990), who state that criminal potential results from several factors early in life, including poor attachment, and that criminal propensity remains relatively stable over time. Our finding is more consistent with the theory of Sampson and Laub (2005), which assumes that correlates of delinquency may change during the life-course. According to Sampson and Laub, changes in life circumstances can generate turning points in an individual’s criminal career. Delinquent behavior is inhibited during childhood and adolescence by bonds to the family and school. During (young) adulthood, social ties to labor or marriage and other turning points in life can modify trajectories of criminal offending.

Several limitations of the present meta-analysis should be noted. First, for reasons of comparability, we only included studies reporting bivariate associations. Multivariate analyses give insight into the unique contribution of attachment to delinquency by simultaneously controlling for other factors. In general, attachment to parents was significantly linked to delinquency in these studies (e.g. Bernburg and Thorlindsson 1999; Ford 2005; Laundra et al. 2002; LeBlanc 1994) or attachment was even the most important predictor of delinquency (e.g., compared to other family factors and economic factors, Mack et al. 2007), while some studies found only weak links between social bonding and delinquency (e.g., compared to prior delinquency and delinquent peers, Agnew 1991). We found 23 manuscripts reporting only multivariate results. Meta-analyzing multivariate associations, however, is problematic, because the effect size statistics of interest depend on other variables in the multivariate model (Lipsey and Wilson 2001, p. 69). The effect sizes derived from multivariate models with different control variables are therefore not comparable with each other and also not comparable with effect sizes derived from bivariate analyses. Moreover, conducting moderator analyses could result in artifactual findings if study characteristics that are tested as moderators would have been used as control variables in the multivariate models of primary studies from which effect sizes are included in the meta-analysis.

Second, this meta-analysis is about *associations* between parental attachment and delinquency, which limits the causal interpretation of our study findings. Although it can be derived from attachment theory that poor attachment can lead to delinquency (Bowlby 1944; Hirschi 1969), research has shown that parents do not only influence their children and the attachment relationship, but that parent-child relationships develop as a result of complex interactions between parents and children (e.g., Crouter and Booth 2003; Granic 2000; Holden 1997). Thus, relationships with parents may also have been affected by the individual characteristics of youngsters, which does not allow drawing conclusions about the direction of the effect. Also, the main aim of this study was to examine the association between attachment and delinquency, however, it would be interesting to know which insecure attachment patterns are most strongly linked to delinquency. Surprisingly, only five studies distinguished between insecure patterns of attachment. More work needs to be done on this issue.

Despite these limitations, this meta-analysis has several strengths. This study connected two areas of research by focusing on empirical studies that have tested hypotheses drawn from social control theory and assumptions from attachment theory. The results were consistent across disciplines. Second, applying a multi-level model made it possible to include all effect sizes with their unique potentially moderating characteristics in the analysis, at the same time correcting for statistical dependency. Third, this meta-analysis tested specific hypotheses drawn from the literature with regard to moderating effects of gender and age rather than testing a series of moderators that are not theory driven as is common in meta-analytic investigations, but increases the risk of chance capitalization. Finally, focusing on a set of limited theoretically based moderators made it possible to build a multivariate moderation model in which the main and interaction effects of age and gender were analyzed, controlling for methodological moderators.

In conclusion, this meta-analysis confirmed that attachment to parents is linked to delinquency in boys and girls. We found several sex differences and age effects: stronger attachment-delinquency links were found in same-sex parent-child pairs compared to cross-sex pairs, and the attachment-delinquency link was stronger for younger participants compared to older participants. These findings suggest that attachment to parents is a viable target for interventions aimed at reducing or preventing delinquency. To be more specific, boys could especially benefit from interventions that focus on relationships with their father, whereas delinquent girls could especially benefit from interventions that focus on the attachment relationship with their mother. When carrying out (preventive) interventions for young delinquents or children at risk for delinquency, one should consider the attachment relationship with parents as a target of intervention. Next to insecure attachment or week bonds to parents these interventions should also aim to improve discipline techniques of parents.
